# NFκB inhibition to lift the mechano-competence of mesenchymal stromal cell-derived neocartilage toward articular chondrocyte levels

**DOI:** 10.1186/s13287-022-02843-x

**Published:** 2022-04-27

**Authors:** Janine Lückgen, Elisabeth Raqué, Tobias Reiner, Solvig Diederichs, Wiltrud Richter

**Affiliations:** 1 Research Centre for Experimental Orthopaedics, Heidelberg University Hospital, Schlierbacher Landstrasse 200a, 69118 Heidelberg, Germany; 2 Department of Orthopaedic and Trauma Surgery, Heidelberg University Hospital, Heidelberg, Germany

**Keywords:** Mesenchymal stromal cells (MSC), Chondrocytes, Mechanical loading, NFκB, Extracellular matrix (ECM) synthesis, Tissue engineering, Mechano-competence, Prostaglandin E_2_ (PGE_2_), Nitric oxide (NO)/iNOS, BMP

## Abstract

**Background:**

Fully functional regeneration of skeletal defects by multipotent progenitor cells requires that differentiating cells gain the specific mechano-competence needed in the target tissue. Using cartilage neogenesis as an example, we asked whether proper phenotypic differentiation of mesenchymal stromal cells (MSC) into chondrocytes in vitro will install the adequate biological mechano-competence of native articular chondrocytes (AC).

**Methods:**

The mechano-competence of human MSC- and AC-derived neocartilage was compared during differentiation for up to 35 days. The neocartilage layer was subjected to physiologic dynamic loading in a custom-designed bioreactor and assayed for mechano-sensitive gene and pathway activation, extracellular matrix (ECM) synthesis by radiolabel incorporation, nitric oxide (NO) and prostaglandin E_2_ (PGE_2_) production. Input from different pathways was tested by application of agonists or antagonists.

**Results:**

MSC and AC formed neocartilage of similar proteoglycan content with a hardness close to native tissue. Mechano-stimulation on day 21 and 35 induced a similar upregulation of mechano-response genes, ERK phosphorylation, NO production and PGE_2_ release in both groups, indicating an overall similar transduction of external mechanical signals. However, while AC maintained or enhanced proteoglycan synthesis after loading dependent on tissue maturity, ECM synthesis was always significantly disturbed by loading in MSC-derived neocartilage. This was accompanied by significantly higher *COX2* and *BMP2* background expression, > 100-fold higher PGE_2_ production and a weaker SOX9 stimulation in response to loading in MSC-derived neocartilage. Anabolic BMP-pathway activity was not rate limiting for ECM synthesis after loading in both groups. However, NFκB activation mimicked the negative loading effects and enhanced PGE_2_ production while inhibition of catabolic NFκB signaling rescued the load-induced negative effects on ECM synthesis in MSC-derived neocartilage.

**Conclusions:**

MSC-derived chondrocytes showed a higher vulnerability to be disturbed by loading despite proper differentiation and did not acquire an AC-like mechano-competence to cope with the mechanical stress of a physiologic loading protocol. Managing catabolic NFκB influences was one important adaptation to install a mechano-resistance closer to AC-derived neocartilage. This new knowledge asks for a more functional adaptation of MSC chondrogenesis, novel pharmacologic co-treatment strategies for MSC-based clinical cartilage repair strategies and may aid a more rational design of physical rehabilitation therapy after AC- versus MSC-based surgical cartilage intervention.

**Supplementary Information:**

The online version contains supplementary material available at 10.1186/s13287-022-02843-x.

## Background

Throughout an organism’s development and life, its tissues are constantly exposed to diverse mechanical stimuli. The cells of the musculoskeletal system function to maintain tissue homeostasis and allow for nondestructive movement of the body even under strenuous physical conditions. Cellular response to mechanical forces is closely related to cell differentiation [1, 2], tissue physiology [3, 4] and pathology [5–7], playing an important role in various musculoskeletal disease states, such as osteoarthritis and osteoporosis. The physical environment of cells varies dependent on localization in cartilage, bone or tendon, and appropriate cell reactions to the experienced mechanical challenge differ considerably between these tissues. Progenitor cells with multilineage differentiation capacity will have to acquire specific mechanical competences needed to respond adequately to the physical stress in the future target tissue. This implies that chondrocytes will likely need a different adaptation than tenocytes or osteocytes. But how do cells develop the appropriate mechano-competence during development? This question is of special importance when progenitor cells like mesenchymal stromal cells (MSC) are used as a therapeutic strategy to stimulate tissue regeneration. In order to reach full tissue functionality, skeletal defects should not only be filled with a similar kind of new tissue, but the regenerating cells should also react to mechanical challenge like their native counterparts. While this works well during bone healing due to constant tissue remodeling, cartilage does not heal to the same extent. Cartilage repair tissue following cell-based therapies like microfracturing and cell implantation displays inferior stiffness and reduced mechanical long-term endurance indicating that mechanical aspects are not properly restored [8, 9]. The reasons for this unsatisfying long-term outcome are still unclear, and it is not known whether a misguided or insufficient mechano-competence of the defect-filling cells is part of the problem.

The main tasks of articular chondrocytes (AC) in native cartilage are to keep the physiological balance and replenish molecules lost from the proteoglycan- and collagen type II-rich extracellular matrix (ECM) during the course of dynamic loading [3]. Cells react and manage load-induced cell stress and ECM alterations by adjustment of their gene expression and metabolism. Thus, important aspects of the mechano-competence of chondrocytes are the capacity to adequately regulate mechano-sensitive genes and pathways to respond to joint-typical dynamic forces, and to support cartilage ECM production to maintain tissue homeostasis. But will proper phenotypic differentiation of MSC into chondrocytes, judged by marker gene expression of *COL2A1* and *ACAN* and the deposition of a cartilage-like ECM, install the proper mechano-competence of native AC?

Surprisingly little is known on the mechano-competence of MSC-derived chondrocytes. Most in vitro studies applying mechanical loading to MSC-based tissue engineering constructs investigated whether load can enhance the efficiency of chondrogenic conversion to replace essential pro-chondrogenic factors like transforming growth factor (TGF)-β [10–16]. Alternatively, it was attempted to reproduce the physical microenvironment in the joint to increase the mechanical stability of MSC-derived neocartilage [17–19]. However, the highly relevant question, whether successful chondrogenesis will automatically teach MSC-derived chondrocytes the proper biological loading response and essential mechano-competence of native AC has so far remained unclear and studies linking cell differentiation with the mechano-biology of MSC and AC are needed.

Since mechanical disuse and overuse regimens result in detrimental effects on cartilage [5], both should be avoided in cartilage regeneration strategies. Thus, it is essential that more knowledge on the mechano-competence of cells used for cartilage therapies is available in order to provide important new measures for evaluation of cartilage tissue regeneration. If native AC and MSC-derived chondrocytes differ in their biological mechano-competence, joint-reloading regimens via continuous passive motion or the timing and intensity of rehabilitation-based physical therapy may need adaptation depending on which cells are implanted or attracted into focal cartilage defects. More knowledge upon which cell adaptation during chondrogenesis allows acquisition of the right mechano-competence also promises the development of better differentiation protocols for tissue engineering and/or pharmacologic co-treatment strategies to optimize patient outcome after cartilage regeneration therapy.

One major difficulty in studying the biological loading response has been the complexity in determining the precise nature of mechanical “signals” perceived by cells in vivo. For example, simple mechanical loading of tissues results in complex physical environments that consist of time-varying stress, strain, fluid flow and pressure, and potentially, other biophysical changes such as osmotic pressure or electric fields generated by the ubiquitous presence of fixed and mobile electric charge on biological molecules [20]. In an in vivo context, mechanical parameters can hardly be controlled. Thus, for a systematic comparison of the mechano-competence of chondrocytes, mechanical challenge of well-designed tissue-engineering constructs in bioreactors under standardized conditions holds great potential. We designed osteochondral units composed of an AC-seeded collagen carrier attached to a porous β-TCP bone replacement material and subjected the neocartilage layer to physiologic dynamic loading in a custom-designed bioreactor imitating 3 h of normal walking in 10-min intervals in a knee joint [21–23]. Global changes in gene expression in response to loading and mechano-sensitive indicator molecules were defined and typical mechano-sensitive pathways activated in AC were determined [21]. Data revealed that loading nascent neocartilage too early disturbed its maturation process while a later application of the same loading protocol maintained metabolic tissue balance and enhanced cartilage matrix production [22]. Whether MSC-derived chondrocytes display the same mechano-competence as AC, such as regulating identical mechano-sensitive targets and pathways and acquiring an equivalent capacity for anabolic loading responses remains open.

The aim of this study was to determine whether MSC-derived chondrocytes acquire the biological mechano-competence of native AC during maturation into neocartilage, and whether they enhance cartilage ECM production after mechanical challenge in correlation with tissue maturation. Our approach was to compare the mechano-competence of human MSC- and AC-derived neocartilage during differentiation for up to 35 days. This new knowledge and the extraction of reasons for potential differences may lead to mechano-adapted redifferentiation protocols and novel pharmacologic co-treatment strategies to optimize clinical cartilage regeneration outcome as well as allow for a more rational design of physical rehabilitation therapy after AC versus MSC-based surgical cartilage intervention.

## Materials and methods

### Isolation and expansion of AC and MSC

Human bone marrow aspirates and articular cartilage samples were obtained from 20 patients (11 female and 9 male, age range 24–92 years, mean age 59 ± 18 years) undergoing total joint replacement surgery with informed written consent of the patients. The study was approved by the local ethics committee on human experimentation of the Medical Faculty of Heidelberg and in agreement with the Helsinki Declaration of 1975 in its latest version. MSC were isolated from bone marrow aspirates by Ficoll Paque Plus density gradient centrifugation as described previously [24]. The mononuclear fraction was washed with phosphate-buffered saline (PBS) and expanded for three passages in medium consisting of high glucose-containing Dulbecco’s modified Eagle’s medium (DMEM, Gibco, Life Technologies, Germany) supplemented with 12.5% fetal bovine serum (FBS, Sigma), 2 mM L-glutamine, 1% non-essential amino acids, 0.1% β-mercaptoethanol (all from Gibco, Life Technologies, Germany), 1% penicillin/streptomycin (Biochrom, Germany) and 4 ng/mL of recombinant fibroblast growth factor-2 (FGF-2) (Miltenyi Biotec, Germany). MSC were seeded in a density of 5,000 cells/cm^2^ and cultured at 37 °C and 6% CO_2_.

Human articular chondrocytes were isolated from macroscopically healthy cartilage areas from 9 patients (6 female and 3 male, age range 56–87 years, mean age 68 ± 9 years) as described previously [25]. After collagenase (1.5 mg/mL)/hyaluronidase (0.1 mg/mL) digestion for 16 h at 37 °C, the cell suspension was filtered to obtain a single cell suspension. AC were expanded in low glucose-containing DMEM with 10% FBS and 1% penicillin/streptomycin for two passages. AC were seeded in a density of 5,700 cells/cm^2^ and cultured at 37 °C and 6% CO_2_.

### Preparation and mechanical stimulation of engineered cartilage tissue

AC or MSC were seeded on both sides of a collagen I/III carrier (diameter 4 mm, height 1.5 mm, Optimaix, Matricel, Germany) at 5 × 10^5^ cells per construct and attached to a block of the bone replacement material β-tricalcium phosphate (β-TCP) (RMS foundation, Switzerland) two days after seeding, building an osteochondral unit as described previously [21]. The constructs were pre-cultured for 3, 21 or 35 days under chondrogenic conditions using high glucose-containing DMEM, 1% penicillin/streptomycin, 0.1 mM dexamethasone, 0.17 mM ascorbic acid-2 phosphate, 2 mM sodium pyruvate, 0.35 mM proline, 5 mg/mL transferrin, 5 ng/mL sodium selenite, 1.25 mg/mL bovine serum albumin (all from Sigma-Aldrich, Germany), 5 mg/mL insulin (Lantus, Sanofi-Aventis, Germany) and 10 ng/mL recombinant human TGF-β1 (Miltenyi or Biomol, Germany). For MSC-derived constructs, insulin, transferrin and sodium selenite were replaced by ITS + premix (Corning, Germany). Medium was changed three times per week.

The dynamic compression protocol (Additional file 1: Fig. S1A) was designed in a previous study [21]. Briefly, day 21 or day 35 constructs were subjected to a single 3-h cyclic unconfined compression episode in a custom-designed bioreactor (Additional file 1: Fig. S1B, C). Constructs were first compressed by 10% of their thickness to guarantee that the contact to the piston was never lost during cyclic compression. Starting from this 10% static offset, the amplitude of cyclic compression was 25% during the loading intervals (10 min) while maintaining the initial 10% static offset during the break intervals (10 min) when cyclic compression was interrupted (Additional file 1: Fig. S1D). This was controlled by the software Galil Tools. A strain of 25% was selected according to Mosher et al. which describe 20–30% as being physiological strain amplitudes for human articular cartilage [26]. After the end of loading, constructs were washed with PBS and subjected to metabolic labeling as described below or snap-frozen in liquid nitrogen and stored at – 80 °C for further analyses.

Where indicated, the chondrogenic medium was supplemented with 100 ng/mL recombinant hBMP6 (starting 1 h before loading, R&D, USA), 500 nM LDN212854 (LDN21, starting at 21 h before the 3-h loading episode, Sigma-Aldrich, Germany), 0.75 µM Bay11-7082 (Bay11) to inhibit NFκB (starting at 45 h before the 3-h loading episode, Sigma-Aldrich, Germany). All treatments were continued during loading and subsequent metabolic labeling of 24 h. Where appropriate, controls were treated with DMSO. For NFκB activation, samples were treated with 10 ng/mL human recombinant IL1-β (Promocell, Germany) in chondrogenic differentiation medium for 3 h.

### Metabolic labeling

Proteoglycan and collagen synthesis were assessed after the end of loading as described previously [22]. After loading, the cartilage layer was detached from the β-TCP block, placed on a nylon mesh in a 48-well plate and labeled with 4 μCi ^35^S as Na_2_SO_4_ or 5 μCi L-[2,3,4,5-^3^H]-proline (both from Hartmann Analytic) for 24 h in 500 μL chondrogenic medium with or without the respective inhibitors or agonists. After washing five times with unlabeled 1 mM Na_2_SO_4_ or 1 mM L-proline in PBS for 20 min while shaking, constructs were digested with 0.5 mg/mL proteinase K at 60 °C and 800 rpm overnight. Incorporated radiolabel was quantified in tissue lysates by β-scintillation counting (1,414 Winspectral software) and normalized to the DNA content determined in the same lysates using the Quant iT Pico Green kit (Invitrogen, Germany).

### Analysis of GAG/DNA and tissue hardness (VLRH)

Total GAG deposition was quantified in proteinase K digested samples using a 1,9-dimethyl-methylene blue (DMMB) assay (Sigma-Aldrich, USA) according to [27] and normalized to the DNA content determined by Quant iT PicoGreen assay according to manufacturer’s instructions (Invitrogen, Germany).

Unconfined indentation testing of osteochondral constructs was performed using the very low rubber hardness (VLRH)-test method at DIN ISO27588 (Digitest II, Bareiss) as described before [28]. Sample hardness was calculated as VLRH units from the depth D that a ball indenter reaches after initial 5 s with a contact force of 8.3 mN and subsequent 30 s with a 100 mN test force as VLRH = 100–0.1D.

### Total RNA isolation and mRNA expression analysis

Total RNA was isolated from the cartilage layer after thawing and mincing by phenol/guanidine isothiocyanate extraction (peqGOLD Trifast, Peqlab, Germany). For mRNA analysis by quantitative real-time PCR (qRT-PCR), total RNA was purified by Zymoclean™ Gel DNA Recovery Kit according to manufacturer’s instructions and reverse transcribed into cDNA using Omniscript reverse transcriptase (Qiagen, Germany) and oligo(dT) primers. Gene expression was assessed by qRT-PCR (Roche diagnostics, Germany). Depicted percentages of reference genes were calculated as 1.8^−ΔCt^ as recommended by the manufacturer of the PCR cycler. ΔCt is the difference between Ct values of the gene of interest and the arithmetic mean of the reference genes *CPSF6* and *HNRPH1*. Primer sequences are listed in Additional file 1: Table S1.

### Western blotting

Frozen osteochondral units were thawed on ice, and the cartilage layer was separated from the β-TCP bone replacement phase and minced in 150 µL PhosphoSafe Extraction Reagent (Merck Millipore, Germany) supplemented with 1 mM Pefabloc® SC (Sigma-Aldrich, Germany) in a mixer mill (Retsch, Germany) at 30 Hz for 2 × 2 min with intermediate cooling on ice for one minute. Subsequently, samples were centrifuged at 13,000×*g* for 20 min at 4 °C to remove cellular debris. Cell lysates were separated by denaturing sodium-dodecyl sulfate polyacrylamide gel electrophoresis and blotted on a nitrocellulose membrane (GE Healthcare, Amersham, Germany) following standard protocols. To detect several proteins of interest in the same lysates, the membrane was cut horizontally at 50 kDa when applicable. The membranes were probed with mouse anti-β-actin antibody (clone AC-15; 1:10,000; GeneTex; GTX26276), mouse monoclonal anti-pERK antibody (clone E-4; 1:200; Santa Cruz; sc-7383), rabbit polyclonal anti-EKR1/2 antibody (1:1000; Cell Signaling Technology; 9102), rabbit monoclonal anti-pSMAD1/5/9 antibody (1:250; Cell Signaling Technology; 13,820), rabbit monoclonal SMAD1/5 antibody (1:500, 1:1000; abcam; ab33902, ab40771), rabbit polyclonal anti-SOX9 antibody (1:2000; Merck Millipore; AB5535), rabbit monoclonal anti-pSMAD2 antibody (1:250; Cell Signaling Technology; 3108), rabbit monoclonal anti-SMAD2/3 antibody (1:250; Cell Signaling Technology; 8685). Bands were detected by peroxidase-coupled goat anti-mouse antibody (1:5000; Jackson ImmunoResearch Laboratories) or peroxidase-coupled goat anti-rabbit antibody (1:10,000; Jackson ImmunoResearch Laboratories) and visualized by enhanced chemiluminescence (Roche Diagnostics, Germany, or Advansta, USA). Densitometric evaluation of band intensities was performed using the software Bio1D.

### Nitrite release

Frozen conditioned medium supernatants of osteochondral units were thawed on ice, and nitrite release was measured using the Griess Reagent System (Promega, USA) according to the manufacturer’s protocol. Briefly, nitrite standard dilutions and samples were incubated with the same volume of sulfanilamide solution in a 96-well plate for 5–10 min at room temperature protected from light. N-(1-napthyl)ethylenediamine dihydrochloride (NED) solution was added, and absorbance at 540 nm was measured after 5–10 min incubation in the dark.

### PGE_2_ release

Frozen conditioned medium supernatants of individual osteochondral units or pooled supernatants of 5 pellets per donor were thawed on ice and analyzed using a competitive PGE_2_ ELISA (Enzo Life Sciences, Switzerland) according to the manufacturer’s protocol. Briefly, PGE_2_ standard dilutions and samples were incubated with an alkaline phosphatase-PGE_2_ conjugate and a monoclonal antibody to PGE_2_ on a goat anti-mouse IgG microtiter plate placed on a plate shaker for 2 h at room temperature. After three washes, the remaining wash solution was removed, and the wells were incubated with a p-nitrophenyl phosphate substrate solution at room temperature for 45 min. Optical density was measured at 405 nm versus 580 nm. Calculated concentrations in constructs were log_2_-transformed.

### Histology and immunohistochemistry

The β-TCP part of the osteochondral units was trimmed to 2–3 mm before decalcifying the samples with Bouin’s solution for two days followed by dehydration in an ascending isopropanol series and embedding in paraffin. After rehydration, proteoglycan deposition was analyzed by staining 5-µm sections with 0.2% (w/v) Safranin O (Fluka, Sigma-Aldrich, Germany) in 1% acetic acid and counterstaining with 0.04% (w/v) Certistain Fast Green (Merck, Germany) in 0.2% acetic acid.

Immunohistochemistry for collagen type II was performed as described [24]. Briefly, 5-µm sections were incubated with 4 mg/mL hyaluronidase in PBS (pH 5.5) and 1 mg/mL pronase (both from Roche Diagnostics, Germany) for antigen retrieval. Sections were blocked with 5% bovine serum albumin (BSA) (Sigma-Aldrich, Germany) and incubated with mouse antihuman collagen type II antibody (II-4C11; 1:1000, ICN Biomedicals, USA). Detection was performed using biotinylated goat anti-mouse secondary antibody, streptavidin alkaline phosphatase (30 min, 20 °C, Dako, Denmark) and fast red (Sigma-Aldrich, USA).

### Statistical analysis

Mean values and standard deviations of independent biological replicates were calculated. Differences between MSC and AC groups were tested using the Student’s two-tailed unpaired *t*-test.

The effect of loading or treatment was assessed within each donor cell population. Cell samples from each donor were tested across treatments (treatment, control) with paired *t*-test. The pairs were defined by the donor cell population. In the case of multiple groups, only the scientifically meaningful group comparisons were assessed using the Student’s two-tailed *t*-test and a post hoc Bonferroni correction to adjust for multiple testing. *P* ≤ 0.05 was considered significant. Data analysis was performed using SPSS 25.

## Results

### Maturation of AC- and MSC-derived neocartilage

**Fig. 1:**
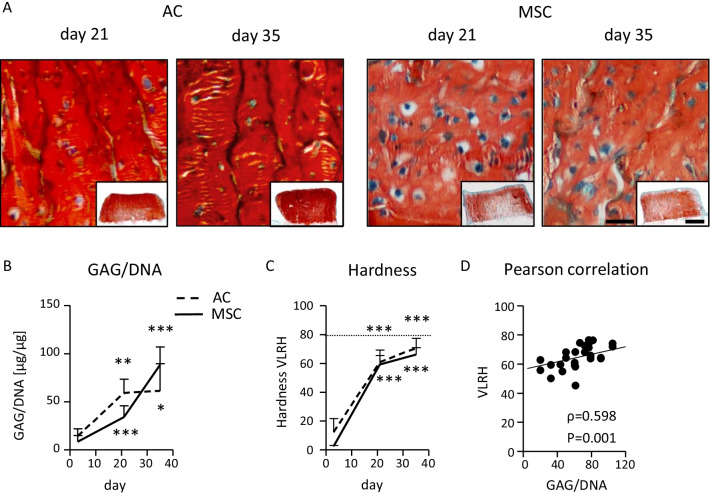
Characterization of AC- and MSC-derived engineered cartilage. Biphasic osteochondral units were seeded with 5 × 10^5^ human articular chondrocytes (AC) or mesenchymal stromal cells (MSC) and cultured for 3, 21 or 35 days under chondrogenic conditions. **A** Standard paraffin sections were stained with Safranin O/Fast Green to visualize GAG deposition (scale bar: 50 μm, overview pictures are presented as insets (scale bar: 1 mm); representative pictures are shown; n = 4-8 donors per group). **B** Mean GAG content ± SD of AC- and MSC-seeded neocartilage measured by DMMB assay, referred to the DNA content (n = 3–10 constructs per time point and group). Two-tailed *t*-test, *p ≤ 0.05, **p ≤ 0.01, ***p ≤ 0.001 in comparison to day 3. **C** Construct hardness over culture time was assessed in VLRH units (n = 3–8 constructs per time point and group). Hardness of native cartilage plugs is indicated as a dotted line. **D** Pearson correlation of VLRH and GAG/DNA (n = 27 samples)

For systematic comparison of the mechano-competence of AC and MSC during neocartilage formation, the pre-culture time for AC- and MSC-seeded osteochondral units was varied between 3, 21 and 35 days. Histological analysis of tissue morphology revealed a chondrocyte-typical round cell morphology on day 21 and 35 in both groups and a strong proteoglycan deposition according to Safranin O staining (Fig. 1A) and collagen type II deposition in neocartilage (Additional file 1: Fig. S2A). The GAG/DNA content of AC- and MSC-derived tissue increased in a similar manner from day 3 to day 35 of maturation (Fig. 1B). In parallel, tissue hardness rose in both groups, as assessed in VLRH units after indentation testing with a metal ball (Fig. 1C). Compared to the hardness values of native articular cartilage plugs set to 100%, AC-derived samples started from 15.5 (± 12.1)% of hardness at day 3 and reached 89.3 (± 8.9)% at day 35 while MSC-derived constructs matured from 3.2 (± 0.9)% at day 3 to 83.8 (± 6.0)% by day 35. Tissue hardness of neocartilage samples positively correlated with the GAG/DNA content (Pearson correlation coefficient 0.598, *P* = 0.001, Fig. 1D). Gene expression analysis of *ACAN* and *COL2A1* confirmed increasing cell differentiation over time in both groups (Additional file 1: Fig. S2B, C). Overall, both cell types matured into neocartilage of similar GAG/DNA content and reached a hardness value close to native tissue.

### Regulation of gene expression in response to loading

**Fig. 2:**
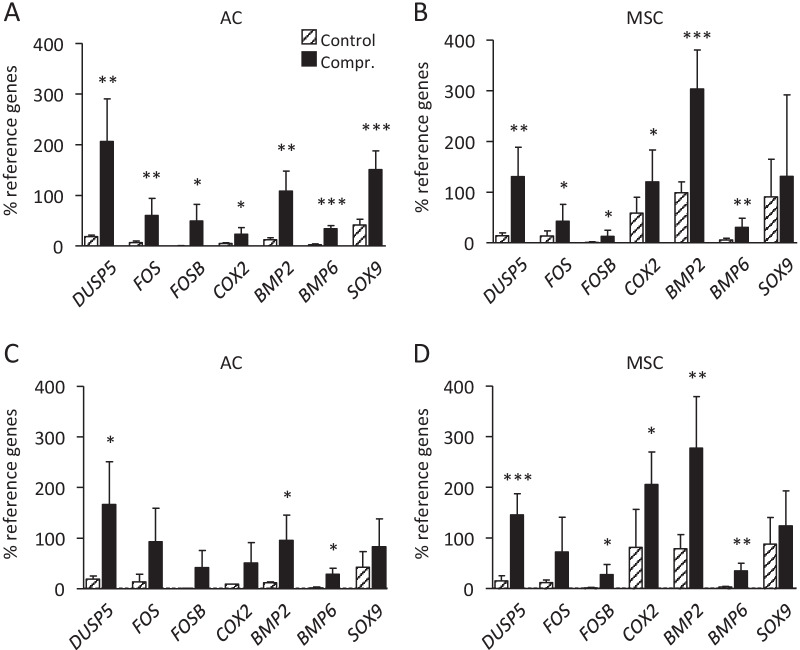
Load-induced regulation of mechano-sensitive gene expression. RNA was isolated at termination of loading from compressed and free-swelling control samples and mRNA levels were determined by qPCR. Gene expression was normalized to the mean expression of reference genes *HNRPH1* and *CPSF6*. **A** AC-derived engineered cartilage on day 21 (n = 5 donors), **B** MSC-derived engineered cartilage on day 21 (n = 8 donors), **C** AC-derived engineered cartilage on day 35 (n = 4 donors), **D** MSC-derived engineered cartilage on day 35 (n = 6 donors). Data are shown as mean ± SD. *T*-test, *p ≤ 0.05, **p ≤ 0.01, ***p ≤ 0.001 compressed vs. free-swelling controls

For comparison of the molecular loading response between AC- and MSC-derived neocartilage, day 3 osteochondral MSC units appeared too soft for a standardized mechanical exposure, possibly due to the more primitive nature of MSC and a virtual absence of cartilage ECM. Thus, regulation of mechano-sensitive genes associated with distinct signaling pathways was determined by qPCR on day 21 and 35. Mechano-sensitive genes associated with the MAPK and BMP pathway were chosen according to results from our previous study [21]. After dynamic loading at both time points, the MAPK signaling pathway-associated factors *DUSP5*, *FOS* and *FOSB* and the BMP molecules *BMP2* and *BMP6* were significantly upregulated in both groups (Fig. 2A–D). Interestingly, before the start of loading, MSC samples showed a higher basal expression of *BMP2* and *COX2* compared to AC at day 21 and 35 while apparent differences in *SOX9* did not reach statistical significance (Additional file 1: Fig. S3A, B). After mechano-stimulation, *COX2* and *BMP2* levels remained significantly higher in MSC-derived chondrocytes compared to AC due to a comparable stimulation in both groups. Altogether, transcriptional regulation of known mechano-sensitive genes was similar in MSC-derived chondrocytes and AC after medium and long-term pre-differentiation. However, MSC-derived chondrocytes responded to loading on a background of significantly higher *COX2* and *BMP2* expression, two interconnected genes located downstream of NFκB signaling [29, 30].

### Activation of mechano-sensitive signaling pathways

**Fig. 3:**
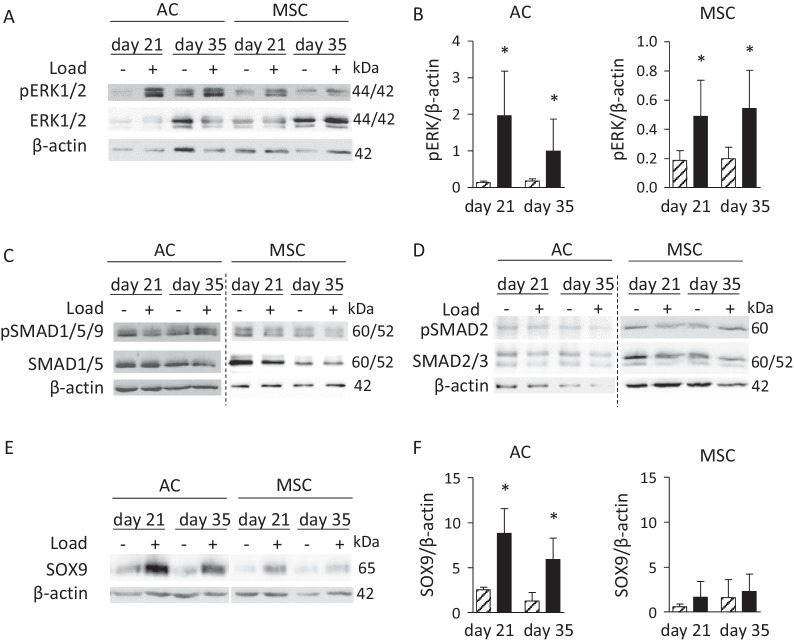
Load-induced regulation of ERK1/2, TGF-β and BMP activity and determination of SOX9 protein. Protein lysates from AC- and MSC-derived cartilage on day 21 and day 35 were subjected to Western blotting. **A** Phosphorylated ERK1/2 and total ERK1/2. **B** Densitometric analysis of pERK Western blot bands normalized to β-actin bands (hatched bar: control, black bar: compressed; *p ≤ 0.05, Mann– Whitney U test vs. control). **C** Phosphorylated SMAD1/5/9 and total SMAD1/5. **D** Phosphorylated SMAD2 and total SMAD2/3. **E** SOX9. **F** Densitometry of SOX9 Western blot bands normalized to β-actin bands (hatched bar: control, black bar: compressed; *p ≤ 0.05, Mann–Whitney U test vs. control). β-actin was used as loading control. Dashed lines indicate separate blots. Representative results from 3–4 donors are depicted

Since MAPK-related target gene expression was load sensitive, we compared regulation of the prominent MAPK mechano-transduction pathway ERK1/2 in AC and MSC groups related to free-swelling controls. Phosphorylated ERK1/2 (pERK1/2) levels rose by loading in a consistent manner in both groups at both time points (Fig. 3A, B) indicating a similar transduction of external mechanical signals into the cells via this pathway. To follow-up on mechano-induction of *BMP2* and *BMP6* expression, BMP pathway activity was assessed by Western blot detection of pSMAD1/5/9 levels in loaded versus non-loaded samples. No consistent load-related regulation of BMP pathway activity was evident in the groups at day 21 and 35 (Fig. 3C), and a similar outcome was observed for TGF-β-associated pSMAD2/3 levels (Fig. 3D). However, the BMP downstream target SOX9 was significantly induced by loading on the protein level in AC at both time points, while upregulation was less consistent and often weaker in MSC-derived osteochondral units (Fig. 3E, F). Overall, 2 of 4 (day 21) and 3 of 4 (day 35) investigated MSC donor populations reacted with some load-induced stimulation of SOX9. Since SOX9 is an important master inducer of cartilage extracellular matrix gene expression, we next asked whether this discrepancy in SOX9 regulation may translate into an altered capacity for ECM production between AC and MSC-derived chondrocytes in response to loading.

### Effects of loading on ECM synthesis

**Fig. 4:**
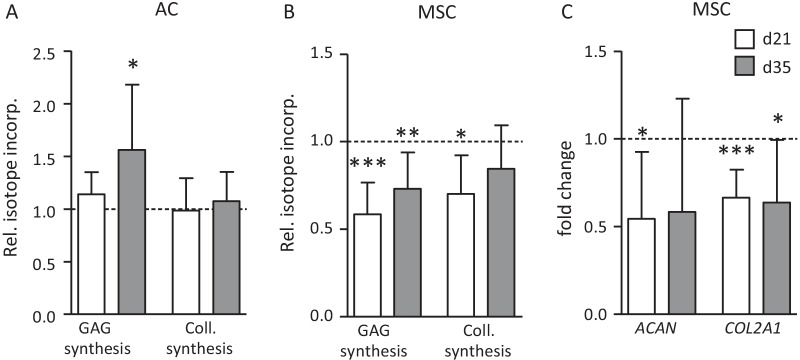
Load-induced alteration of GAG and collagen synthesis and expression of differentiation markers. **A**, **B** Changes in ^35^S-sulfate and ^3^H-proline incorporation over 24 h following termination of loading on day 21 (d21) and day 35 (d35) are shown. Data were normalized to the DNA content of samples and values of the non-loaded controls were set to 1 (dashed line). **C** Effects of loading on *ACAN* and *COL2A1* gene expression in MSC-based neocartilage at day 21 and day 35. Data of compressed samples are shown as mean ± SD relative to the uncompressed group indicated by a dashed line. *T*-test, *p ≤ 0.05, **p ≤ 0.01, ***p ≤ 0.001 compressed versus free-swelling controls (n = 3–10 constructs)

To compare the effects of the chosen anabolic loading episode on ECM synthesis in AC- and MSC-derived cartilage, de novo synthesis of GAG was determined by ^35^SO_4_ incorporation over 24 h after termination of loading on day 21 and 35. In separate samples, collagen synthesis was assessed alike by ^3^H-proline incorporation and both values were normalized to the DNA content of samples. On day 21, loading disturbed neither GAG synthesis nor collagen synthesis in AC-derived cartilage as described previously [22]. On day 35, GAG synthesis was significantly enhanced while collagen synthesis was maintained in AC-derived neocartilage (Fig. 4A). Opposite to this beneficial outcome of loading in the AC group, GAG/DNA synthesis was significantly inhibited by 41 (± 17)% (*P* = 0.00001) after loading of MSC-derived neocartilage on day 21 and by 27 (± 20)% on day 35 (*P* = 0.003). Furthermore, collagen synthesis was significantly reduced (*P* = 0.016) on day 21 and downregulated by trend on day 35 (Fig. 4B). Negative effects of loading on MSC-derived chondrocytes were also evident from a significantly downregulated gene expression of *ACAN* and *COL2A1* (Fig. 4C) while both molecules remained unaltered after loading of AC ([22] and data not shown). Thus, while AC maintained or enhanced ECM synthesis upon exposure to dynamic loading dependent on tissue maturity, ECM production was significantly disturbed by loading in MSC-derived neocartilage also after extended pre-maturation. Overall, this demonstrated that well-differentiated MSC-derived chondrocytes did not acquire a mechano-competence like AC to cope with the mechanical stress of a physiologic loading protocol, but rather showed a higher vulnerability to be disturbed in ECM production by loading.

### BMP stimulation and differential regulation of ECM synthesis

**Fig. 5:**
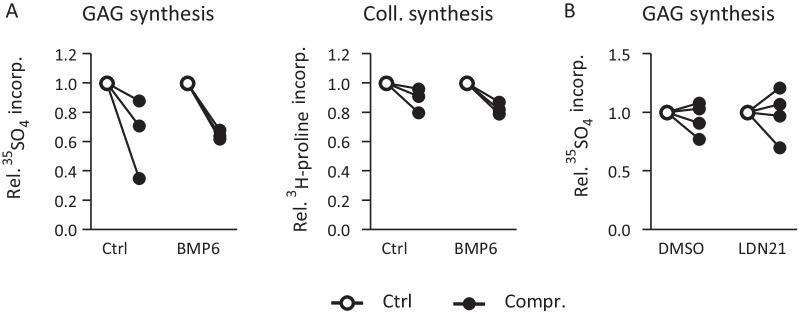
Influence of the BMP pathway on load-induced alterations of ECM synthesis. **A** MSC-seeded osteochondral units pre-cultured for 21 days were treated with 100 ng/mL recombinant BMP6 starting 1 h before loading, during loading (or free-swelling) and during 24 h of radiolabel incorporation. Changes in ^35^S-sulfate and ^3^H-proline incorporation per DNA are depicted relative to the free-swelling control set to 1 (n = 3 donors). **B** AC-seeded osteochondral units pre-cultured for 21 days were treated with 500 nM of the BMP receptor (ALK1/2/3) inhibitor LDN-212854 (LDN21) or DMSO beginning 21 h before start of loading, during loading and during radiolabel incorporation. Alterations in ^35^S-sulfate incorporation per DNA are depicted relative to the free-swelling control set to 1 (n = 4 donors)

The consistent induction of the BMP-responsive SOX9 protein by loading only in AC-derived cartilage along with better ECM synthesis suggested that an anabolic pathway like the BMP pathway may be of relevance for the better mechano-competence of AC versus MSC-derived chondrocytes. Thus, we tested whether stimulation of MSC-derived neocartilage by exogenous BMP is a means to rescue the negative regulation of ECM synthesis in MSC-derived chondrocytes in response to loading. MSC-derived neocartilage was exposed to 100 ng/ml exogenous BMP6 on day 21 1 h before start of compression, during loading and during radiolabel incorporation (24 h after end of loading). BMP6 treatment did not eliminate the negative effects of loading on GAG synthesis in MSC-derived cartilage nor was a different response seen for collagen synthesis (Fig. 5A). To challenge the relevance of BMP signaling for ECM synthesis in AC-derived cartilage, the BMP pathway was inhibited by 500 nM of LDN21 on day 21 starting from 21 h before start of loading. Inhibition was maintained during loading and during isotope incorporation over 24 h after end of loading. Despite inhibition of BMP signaling by LDN21, AC-derived engineered cartilage still showed undisturbed GAG synthesis after loading (Fig. 5B). Together with data from the MSC group this demonstrated, that BMP pathway activity was not rate limiting for ECM synthesis after loading and could not explain the recorded differential response of AC and MSC-derived chondrocytes. Therefore, next focus was on catabolic influences which may be related to the differential loading response.

### Catabolic factors and differential regulation of ECM synthesis

Among known catabolic factors disturbing the metabolic balance and ECM synthesis of chondrocytes, nitric oxide (NO), produced by iNOS, and prostaglandin E_2_ (PGE_2_), synthesized under contribution of COX2, have been shown to be mechano-regulated in chondrocytes in previous studies [31–34]. Beyond, the NFκB pathway was repeatedly associated with negative effects on chondrocyte ECM synthesis and the degradation of cartilage in the course of OA [35, 36]. Thus, we addressed the relevance of all three candidates for the loading response of AC- versus MSC-derived neocartilage on day 21.

**Fig. 6:**
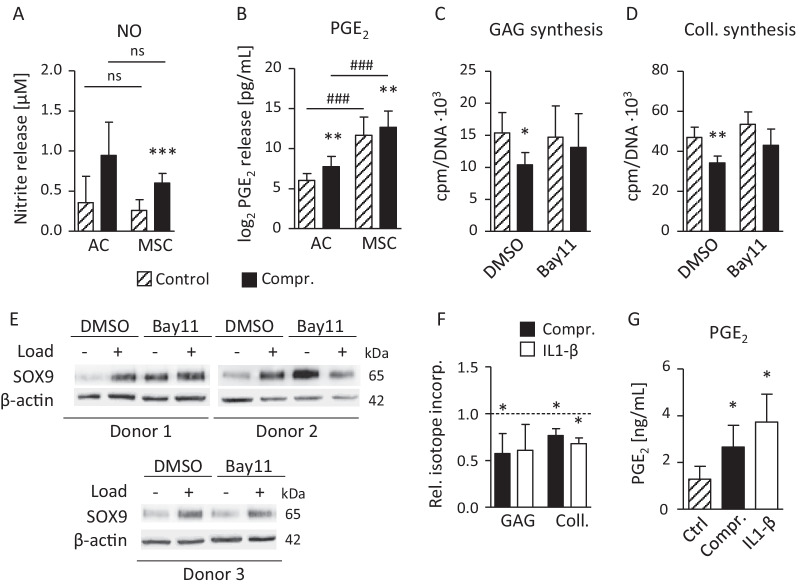
Load-induced release of NO, PGE_2_ and relevance of NFκB for SOX9 and ECM synthesis. Conditioned medium supernatant (20 h) from AC- and MSC-derived neocartilage compressed on day 21 and from control samples was analyzed for **A** nitrite concentration by the Griess test (n = 6–9 per group) and **B** PGE_2_ content by ELISA. PGE_2_ data were log_2_ transformed to cope with the more than 100-fold different levels of AC- and MSC-derived samples (n = 6–9). **C**, **D** MSC-derived cartilage was treated with 0.75 µM of the IκBα phosphorylation inhibitor Bay11-7082 (Bay11) or DMSO starting 45 h before beginning of loading on day 21. Changes in GAG synthesis per DNA were determined by ^35^S-sulfate incorporation and changes in collagen protein synthesis per DNA were determined by ^3^H-proline incorporation over 24 h following termination of loading (n = 4). **E** Regulation of SOX9 protein levels in MSC-derived neocartilage treated by the NFκB inhibitor or DMSO and subjected to loading was determined by Western blot analysis using β-actin as a loading control. **F** MSC-derived cartilage pre-cultured for 21 days was subjected to dynamic loading (3 h) or non-loaded samples were instead treated with 10 ng/ml IL1-β for 3 h before changes in GAG synthesis and collagen synthesis were determined by ^35^S-sulfate or ^3^H-proline incorporation, respectively. Values were normalized to the DNA content and data are depicted relative to non-loaded and non-treated samples set to 1 (dashed line, n = 3–4 donors). **G** PGE_2_ content in conditioned medium supernatants (20 h) was determined by ELISA in controls and after 3 h of cyclic compression or stimulation with 10 ng/mL IL1-β (n = 3–4). *T*-test with Bonferroni correction, *p ≤ 0.05, **p ≤ 0.01, ***p ≤ 0.001 compressed vs. controls; #*p* ≤ 0.05, ##*p* ≤ 0.01, ### *p* ≤ 0.001 AC versus MSC

Culture supernatants collected after loading from compressed and free-swelling samples revealed a significant elevation of NO production by loading which was, however, not different between the groups (Fig. 6A). In line with the significantly higher *COX2* expression of MSC-derived neocartilage, PGE_2_, was significantly higher in medium supernatants from free-swelling MSC versus AC samples (mean 138-fold; Fig. 6B). Like *COX2* expression, PGE_2_ release was significantly stimulated by loading in AC-derived (mean 3.3 ± 1.1-fold, *P* = 0.003) and in MSC-derived cartilage (mean 1.9 ± 0.4-fold, *P* = 0.002). This demonstrated for the first time that huge differences exist in prostaglandin production between MSC- and AC-derived engineered cartilage under identical differentiation conditions. To judge influences from the osteochondral design of samples and bioreactor culture, PGE_2_ levels were also determined during a time course of classical AC and MSC pellet differentiation culture (Additional file 1: Fig. S4). Donor variability of PGE_2_ release was high for AC-derived samples on day 7 and while mean PGE_2_ production of AC vanished over time toward the assay detection level (13.4 pg/ml), secretion from MSC-derived neocartilage remained high and significantly elevated compared to AC (73–375-fold). Thus, high PGE_2_ production was a characteristic feature of MSC undergoing chondrogenesis, a finding which may influence the stress response after mechanical loading.

It is widely acknowledged that NFκB signaling is one main catabolic pathway driving ECM degradation in cartilage during OA progression. Thus, we investigated whether NFκB signaling plays a role in the load-induced suppression of ECM synthesis in MSC-derived neocartilage. In order to establish a causal relationship between the loading response and NFκB signaling, MSC-derived neocartilage was exposed to 0.75 µM BAY11-7082 (45 h before, during loading and isotope incorporation), a potent inhibitor of IκBα phosphorylation [37]. Under NFκB inhibition on day 21, the significant drop of GAG synthesis by 31 (± 12)% (*P* = 0.037) after loading was rescued (Fig. 6C), and collagen synthesis was no longer significantly suppressed as seen in the DMSO-treated control group (Fig. 6D). This is in line with the known negative effects of NFκB signaling on cartilage ECM synthesis [36], and we addressed whether SOX9 protein regulation may be involved in this outcome. Under NFκB inhibition, SOX9 levels rose in free-swelling controls in 2 of 3 experiments (Fig. 6E), but inhibition did not install a stronger SOX9 protein inducibility by loading as this is observed in AC-derived cartilage (Fig. 3E, F). To further challenge the negative role of NFκB signaling on ECM synthesis, MSC-derived cartilage from day 21 was exposed to IL1-β, a prominent activator of the NFκB pathway in human chondrocytes [38]. In fact, 3 h of treatment with 10 ng/mL IL1-β instead of loading mimicked the reduction of GAG and collagen synthesis recorded in the parallel samples subjected to loading (Fig. 6F). Importantly, also PGE_2_ production was significantly enhanced in a similar manner by IL1-β treatment as well as by dynamic loading (Fig. 6G) confirming its inducibility by pro-inflammatory cytokines and mechanical loading. Thus, catabolic NFκB activity influencing SOX9 expression was a rate-limiting parameter for ECM synthesis in MSC-derived neocartilage exposed to loading.

Taken together, our data demonstrated that MSC-derived chondrocytes show enhanced vulnerability to be disturbed by loading and did not acquire a mechano-competence like native AC during classical in vitro differentiation and neocartilage formation. Catabolic influences from NFκB signaling and altered regulation of SOX9 protein levels hindered the sustained ECM production after loading seen in AC, a deficiency that could in part be outbalanced by NFκB pathway inhibition.

## Discussion

Joint surface defects in weight-bearing joints are frequent and often require surgical therapy to provide symptom relief and are an attempt to avoid possible progression into osteoarthritis [39]. Most joint preserving treatment protocols like microfracture or chondrocyte implantation require new cartilage to be formed at the site of injury and, thus, under mechanical conditions which are unfavorable for healthy cartilage formation. Although treatment is initially efficacious in a large number of patients, the average long-term outcome is often unsatisfactory [9, 39, 40]. Newly formed cartilage tissue from these therapeutic strategies lacks the structural organization of native cartilage and has inferior mechanical properties making it prone to failure [8]. The ultimate goal of cartilage regeneration is to develop a replacement tissue which shows not only a structure and composition that resembles native cartilage, but that yields a tissue of similar mechanical competence to fully restore joint functionality. This requires that regenerating cells adopt a similar mechanical behavior to native articular chondrocytes; however, information about a functional mechanical similarity between native chondrocytes and multipotent regenerating cells developing into chondrocytes was so far not available.

MSC reside as skeletal stem cells in bone marrow and invade into cartilage defects from subchondral bone after microfracturing or are implanted after harvest and expansion from bone marrow aspirates. In the current in vitro study we addressed, whether MSC acquire the proper biological loading response of native AC during in vitro development into chondrocytes. We ensured that AC- and MSC-derived neocartilage pre-matured before loading and contained well-differentiated round chondrocytes expressing high levels of *COL2A1* and *ACAN*, reached a similar amount of GAG/DNA and hardness value close to native tissue. Although basic features of mechano-transduction, like activation of the pERK1/2 pathway or regulation of common mechano-sensitive genes, like *BMP2*, *BMP6,* and *COX2*, were similar between AC- and MSC-derived cartilage, physiologic loading disturbed GAG and collagen synthesis only in the MSC group. This negative outcome was independent of the pre-culture period and contrasted the maintained (day 21) or even enhanced ECM synthesis (day 35) after loading observed for AC-derived neocartilage.

Downstream effects of biomechanical signals on engineered cartilage are conditioned by their mode, magnitude, frequency and duration as well as by pre-culture time, microenvironmental cues and the presence of growth factors. The vast variations existing between different studies on human and nonhuman chondrocytes and MSC (reviewed in [16, 41]) usually preclude a direct comparison of the outcome. Thus, the negative [42, 43], neutral [17, 44] or positive effects [18, 45, 46] of compressive loading on chondrogenic MSC differentiation and ECM production reported in diverse studies are not necessarily conflicting. An important novelty of our study is to base conclusions about anabolic or catabolic effects of loading on chondrocytes on a highly sensitive and quantitative measurement of GAG and collagen de novo synthesis and, thus, a direct measurement of the metabolic adaptation of the cells. This allowed us to discover the higher vulnerability of MSC-derived chondrocytes to be disturbed by loading, suggesting that physical rehabilitation protocols may need an adaptation depending on the cell source regenerating the new tissue. We further introduced *BMP2*, *BMP6*, *COX2*, PGE_2_ and NO into the list of mechano-sensitive molecules regulated in MSC-derived cartilage.

The inferior mechano-competence of MSC-derived chondrocytes occurred on a background of significantly higher *BMP2* and *COX2* expression compared to AC. The loading response further lacked the consistently strong induction of SOX9 protein which was observed in AC here and in previous studies [21, 47]. With the aim of enhancing SOX9 levels in MSC-derived cartilage, we stimulated the anabolic BMP pathway by BMP6 treatment during loading, but this did not rescue negative effects of loading on ECM synthesis. Since BMP receptor inhibition during loading was not rate limiting for load-induced ECM synthesis in AC-derived cartilage, this anabolic pathway could not explain the differential loading response of AC- and MSC-derived chondrocytes.

Catabolic processes in cartilage degradation during osteoarthritis include stimulation of NO, PGE_2_ and the NFκB pathway, and we investigated their connection to the mechano-competence of MSC-derived cartilage. In line with previous studies that reported NO stimulation in chondrocytes under cyclic tensile strain [48], hydrostatic pressure [49], fluid flow shear stress [31] or by compression of porcine [32] or mouse cartilage explants [34] as well as chondrocytes cultured in agarose [50], we observed a stimulation of NO production in both AC and MSC-derived chondrocytes under dynamic compression. Importantly, the compression-induced NO release of MSC-derived chondrocytes was similar to AC, indicating NO as a general mechano-responder similar to pERK1/2 and abovementioned mechano-response genes. Of note, this catabolic molecule could not explain the differential loading response of MSC- and AC-derived neocartilage.

PGE_2_ is one of the most important stress-induced factors in skeletal tissues, and the observed induction by mechanical loading is in line with previous studies using fluid flow shear stress on bovine chondrocytes [51] or compression of mouse cartilage explants [34]. Importantly, we demonstrated a strong difference between neocartilage derived from MSC or AC. Accompanied by significantly higher *COX2* expression, MSC-derived tissue released more than two orders of magnitude more PGE_2_, inviting the speculation that COX2/PGE_2_ may reduce the capacity of the cells to maintain ECM synthesis after mechanical loading. In line with this hypothesis, chondrocytes have previously been reported to reduce proteoglycan deposition along *ACAN* expression when high-density or alginate cultures were treated with PGE_2_ [52]. In addition, human cartilage explants reduced proteoglycan synthesis when treated with PGE_2,_ and this could be rescued by a COX2 inhibitor or an PGE_2_ receptor antagonist [53]. On the other hand, a strong body of early publications on classical chick limb bud cultures demonstrated that PGE_2_ stimulates chondrogenesis in mesenchymal cultures [54–58]. More recent data have revealed that PGE_2_ treatment is not affecting GAG production of human MSC during standard chondrogenic 3D pellet culture [59]. Fermor et al. [60] claimed that compression stimulates PGE_2_ only under hyperoxic (20%) but not under normoxic (1%) conditions in porcine cartilage explants. These conflicting data within the literature discouraged us from considering PGE_2_ as the main catabolic factor explaining the higher vulnerability of MSC-derived chondrocytes to loading and instead we focused on NFκB pathway activity. Also contributing to this consideration was the similar GAG and collagen synthesis in free swelling AC- and MSC-derived osteochondral units despite over 100-fold differences in PGE_2_ levels recorded in the current study, and the identical PGE_2_ stimulation due to loading. Nevertheless, future investigations should shed more light on whether and how our discovered drastic differences in PGE_2_ production between AC- and MSC-derived neocartilage may affect their differentiation capacity and stress reaction in response to diverse stimuli.

Importantly, we showed for the first time that inhibition of the catabolic NFκB pathway during cyclic compression installed higher SOX9 protein levels in MSC-derived chondrocytes, and it rescued the negative effects seen after loading on ECM synthesis although GAG and collagen synthesis remained unchanged under free-swelling conditions. This demonstrated that in MSC-derived neocartilage the loading-response involved negative input from NFκB signaling, a pathway which was previously already associated with negative effects on cartilage ECM synthesis [36] and inhibition of SOX9 [61, 62]. In chondrocytes exposed to cyclic dynamic tensile strain of high magnitude (25–50%), nuclear translocation of NFκB and transcriptional activation of pro-inflammatory activators like COX2, IL1-β and TNF-α was observed and in parallel, *COL2A1* and *ACAN* expression was inhibited [63–65]. In agarose constructs seeded with bovine chondrocytes, activation and nuclear translocation of NFκB in response to low magnitude dynamic compression (0–15%) occurred only under IL1-β stimulation [66], indicating a tight interaction between NFκB and pro-inflammatory signals in loaded chondrocytes. For this reason, we tested IL1-β and TNF-α gene expression in response to loading in our model, but both remained below detection level in control and loaded AC- and MSC-derived cartilage (data not shown). This absence of an immediate pro-inflammatory response to cyclic compression in AC underlines the physiologic nature of the chosen loading protocol and reflects no divergent pro-inflammatory input from IL1-β and TNF-α in both groups, although stimulation with exogenous IL1-β had the capacity to suppress ECM synthesis when applied instead of loading.

One limitation of our study is that native AC were derived from patients diagnosed with osteoarthritis, and it cannot be excluded that this may have affected the mechano-response of the cells. We harvested chondrocytes only from macroscopic intact cartilage areas and demonstrated that AC-derived neocartilage reacted to the physiological loading protocol with an anabolic response as it would be expected from healthy cartilage. Nevertheless, unless experiments are repeated with cells from healthy donors, the osteoarthritis background of AC should be kept in mind.

The described differential mechano-response between MSC- and AC-derived neocartilage is currently limited to one dynamic loading protocol which we specifically chose for its ability to evoke an anabolic loading response in AC after sufficient pre-maturation. It will be very interesting to undertake similar comparative studies in the diverse mechanical settings described by other authors, including those reporting stimulation of chondrogenic markers in MSC cultures in response to loading ([14, 15, 18] and reviewed in [16]) and test whether the mechano-response of AC may still surpass MSC-derived chondrocytes under such conditions. This will allow broader results, and it will distinguish general differences between MSC- and AC-derived neocartilage from system- or load-specific influences, thereby significantly deepening our understanding and capability to install a full mechano-competence in MSC-derived neocartilage. Last but not least, the contribution of additional factors like mechano-regulated microRNAs [23] and differentiation-relevant pathways like IGF/PI3K/AKT signaling should be addressed in the future. Large animal studies will be needed to judge the potential of NFκB inhibition after surgical cartilage intervention for enhancing the outcome of MSC-based cartilage regeneration strategies in the less controlled in vivo environment under long-term loading and physiological oxygen conditions.

## Conclusions

In conclusion, we here demonstrated for the first time that well-differentiated MSC-derived chondrocytes producing more than 100-fold more PGE_2_ than AC did not acquire a mechano-competence like native chondrocytes in mature neocartilage, but were more vulnerable to be disturbed by a physiologic loading episode which imitates 3 h of normal walking in a knee joint. Catabolic influences from NFκB signaling hindered the sustained GAG and collagen production after dynamic compression observed with AC-derived neocartilage. Managing catabolic NFκB influences during loading was one important adaptation to enhance the chondrogenic master transcription factor SOX9 and install a mechano-resistance closer to AC-derived neocartilage. These basic insights are highly important to design mechano-adapted differentiation protocols for MSC, novel pharmacologic co-treatment strategies to optimize clinical cartilage regeneration outcome as well as to allow for a more rational design of physical rehabilitation therapy after AC- versus MSC-based surgical cartilage intervention.

## Supplementary Information

**Additional file 1.** Supplementary Figures S1-S4 and Supplementary Table S1.

## Data Availability

The datasets generated during and/or analyzed during the current study are available from the corresponding author on reasonable request.

## Abbreviations

AC: Articular chondrocytes
ACAN: Aggrecan
Bay11: Bay11-7082
BMP: Bone morphogenetic protein
BSA: Bovine serum albumin
COL2A1: Collagen type II alpha 1
COLL: Collagen
COX2: Cyclo oxygenase-2
CPSF6: Cleavage and polyadenylation specific factor 6
Ctrl: Control
D: Depth
DMEM: Dulbecco’s modified Eagle medium
DMMB: 1,9-Dimethyl-methylene blue
DMSO: Dimethyl sulfoxide
(c)DNA: (Complementary) Deoxyribonucleic acid
DUSP5: Dual specificity protein phosphatase 5
ECM: Extracellular matrix
ELISA: Enzyme-linked immunosorbent assay
(p)ERK: (Phosphorylated) extracellular signal-regulated kinase 1/2
FBS: Fetal bovine serum
FGF: Fibroblast growth factor
FOS: Fos proto-oncogene
FOSB: FosB proto-oncogene
GAG: Glycosaminoglycan
HNRPH1: Heterogeneous nuclear ribonucleoprotein H
IgG: Immunoglobulin G
IκBα: Nuclear factor of kappa light polypeptide gene enhancer in B cells inhibitor, alpha
IL1-β: Interleukin 1 beta
iNOS: Inducible nitric oxide synthase
LDN21: LDN-212854
MAPK: Mitogen-activated protein kinase
MSC: Mesenchymal stromal cells
NED: N-(1-Napthyl)ethylenediamine dihydrochloride
NFκB: Nuclear factor "kappa light chain enhancer" of activated B cells
NO: Nitric oxide
OA: Osteoarthritis
PBS: Phosphate-buffered saline
PGE_2_: Prostaglandin E_2_
qRT-PCR: Quantitative real-time polymerase chain reaction
(m)RNA: (Messenger) Ribonucleic acid
Saf O: Safranin Orange
(p)SMAD: (Phosphorylated) Suppressor of mothers against decapentaplegic
SO_4_: Sulfate
SOX9: SRY (sex-determining region Y)-box transcription factor 9
β-TCP: Beta-tricalcium phosphate
TGF-β: Transforming growth factor beta
TNF-α: Tumor necrosis factor alpha
Vs: Versus
VLRH: Very low rubber hardness
