# Supplementary material for: NFκB inhibition to lift the mechano-competence of mesenchymal stromal cell-derived neocartilage toward articular chondrocyte levels
Source: Stem Cell Res Ther. 2022 Apr 27;13:168. doi: 10.1186/s13287-022-02843-x (PMC9044876; doi:10.1186/s13287-022-02843-x)
Supplement: Supplementary file 1 — Additional file 1. Supplementary Figures S1-S4 and Supplementary Table S1. [file 13287_2022_2843_MOESM1_ESM.pdf]

Electronic supplementary material for:

NFκB inhibition to lift the mechano-competence of mesenchymal stromal cell-derived neocartilage toward articular chondrocyte levels

Janine Lückgen[1], Elisabeth Raqué[1], Tobias Reiner[2], Solvig Diederichs[1] and Wiltrud Richter[1*]

*To whom correspondence should be addressed:

E-mail: wiltrud.richter@med.uni-heidelberg.de

This pdf file includes:

Supplementary Fig. S1-S4
Supplementary Table S1

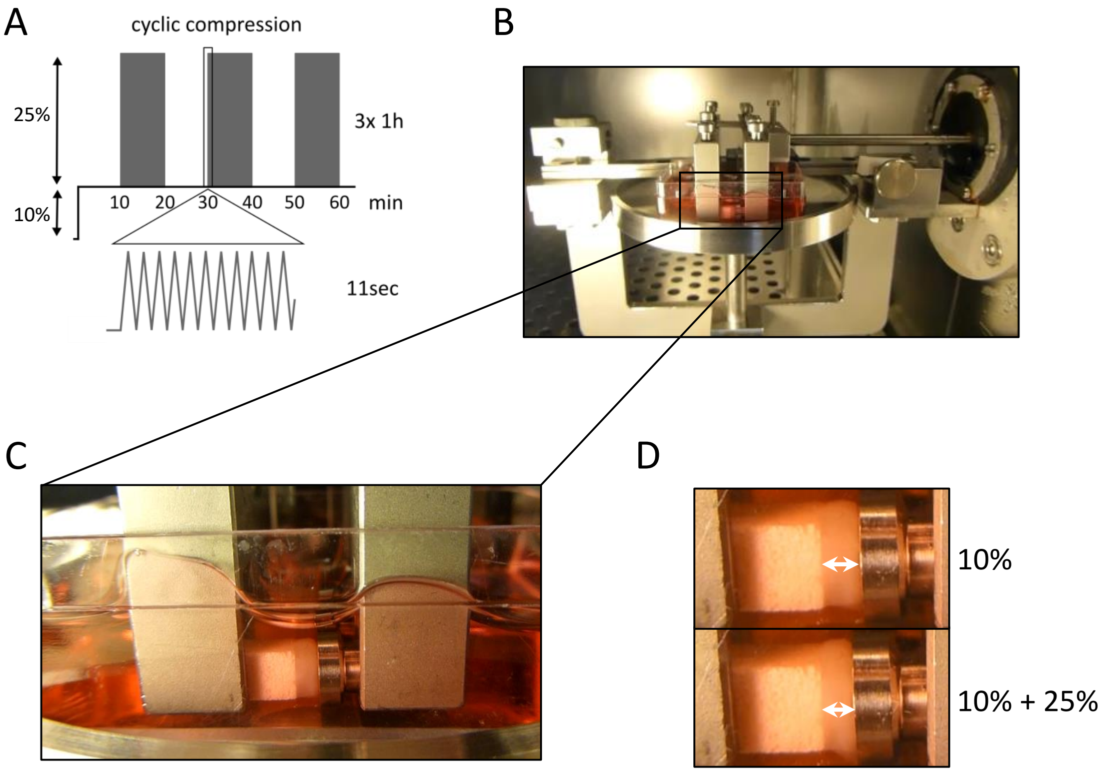

Supplementary Fig. S1. Loading conditions. AC- and MSC-seeded osteochondral units were subjected to a single 3-h cyclic compression episode in a custom-made bioreactor. **(A)** Schematic visualization of the applied loading protocol. **(B)** Representative picture of samples placed in the bioreactor. **(C)** Close-up picture of samples placed in the bioreactor. The piston touches the chondral phase of the osteochondral unit. **(D)** The 10% static offset and the superimposed additional 25% compression is shown for a representative sample.

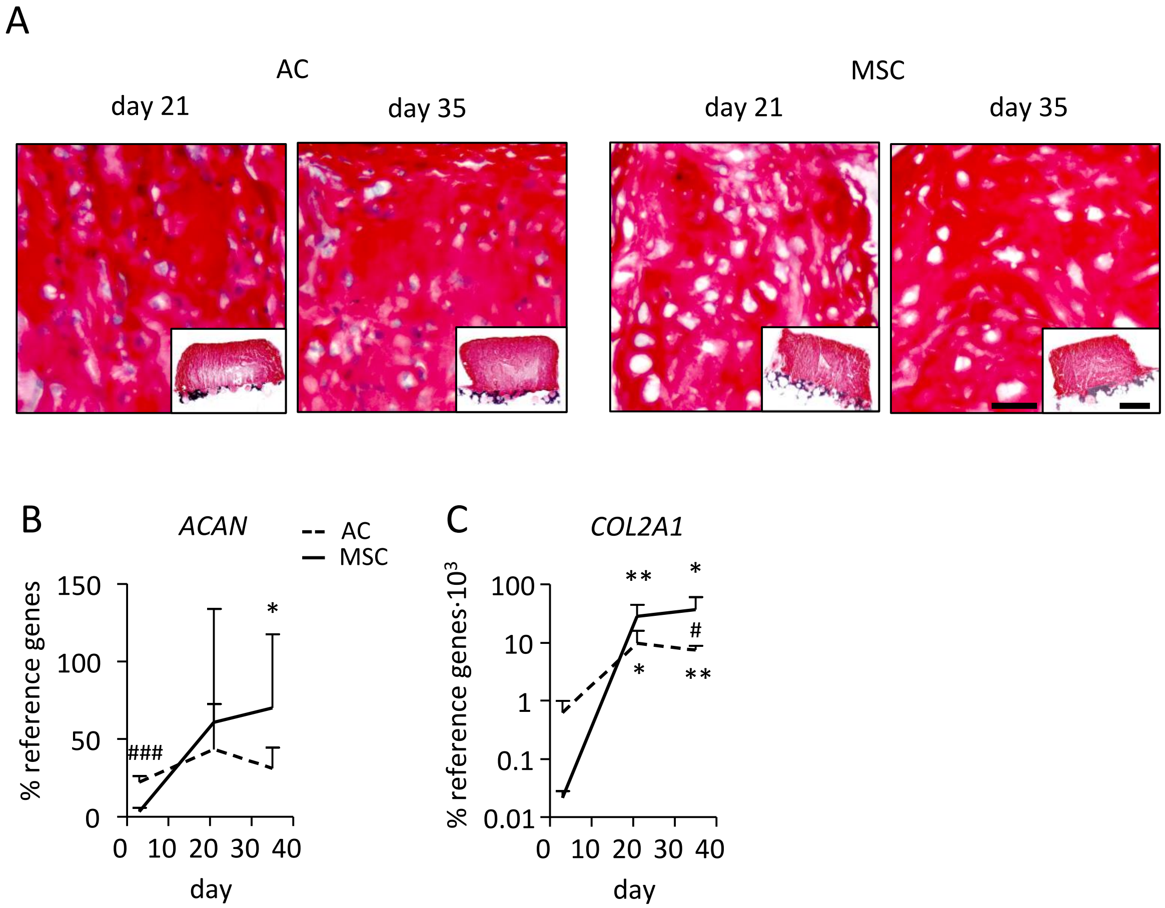

Supplementary Fig. S2. Characterization of AC- and MSC-derived engineered cartilage. Biphasic osteochondral units were seeded with 5×10^5 human articular chondrocytes (AC) or mesenchymal stromal cells (MSC) and cultured for 3, 21 or 35 days under chondrogenic conditions. **(A)** Collagen type II was detected by immunohistochemistry of standard paraffin sections. Black: left-over β-TCP (scale bar: 50 µm (inset: 1 mm); representative pictures are shown; n=4-8 donors per group). **(B, C)** Mean gene expression ± SD is given as percentage of the mean levels of reference genes *HNRPH1* and *CPSF6* (n=3-8 donors). Two-tailed t-test with Bonferroni post-hoc test, $*p \le 0.05$, $**p \le 0.01$, $***p \le 0.001$ in comparison to day 3, $\#p \le 0.05$, $\#\#\#p \le 0.001$ AC vs. MSC at the same time point.

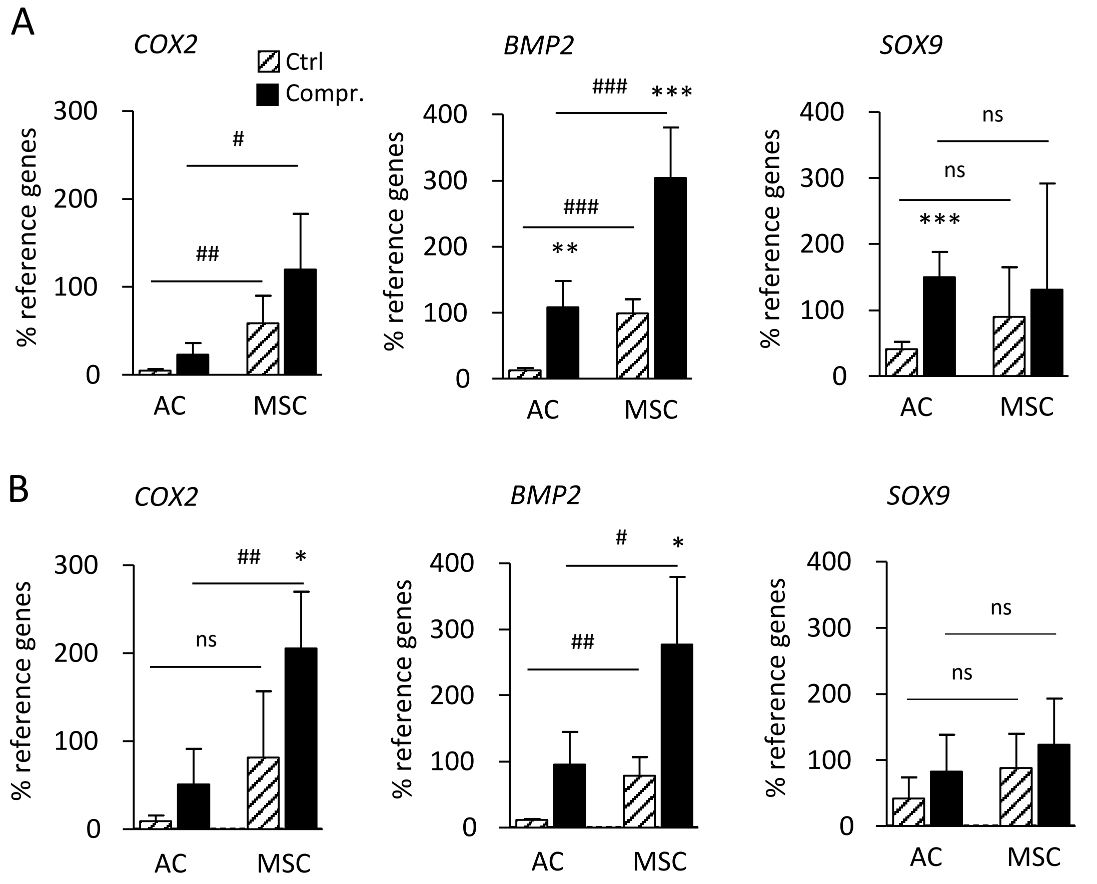

Supplementary Fig. S3. **Comparison of gene expression levels in AC- vs. MSC-derived cartilage.** RNA was isolated at termination of loading of compressed samples and free-swelling controls and gene-specific mRNA levels were determined by qPCR. **(A)** *COX2, BMP2* and *SOX9* expression levels in AC- and MSC-derived cartilage on day 21. **(B)** *COX2, BMP2* and *SOX9* expression levels in AC- and MSC-derived cartilage on day 35. Gene expression was normalized to reference genes *HNRPH1* and *CPSF6*. T-test with Bonferroni correction, *p ≤ 0.05, **p ≤ 0.01, ***p ≤ 0.001 compressed vs. free-swelling controls, #p ≤ 0.05, ##p ≤ 0.01, ###p ≤ 0.001 AC vs. MSC (n=4-8).

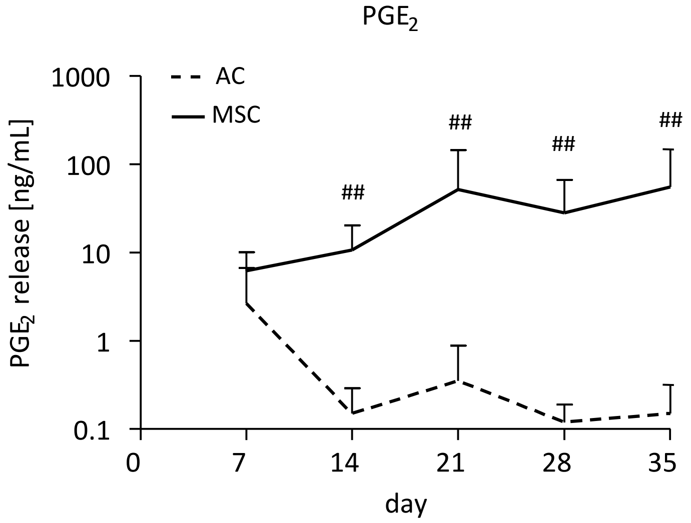

Supplementary Fig. S4. PGE_2 release from AC and MSC pellets cultured under chondrogenic conditions over time. Conditioned medium supernatants (2 days) from AC and MSC-derived chondrocyte pellets were collected weekly and analyzed for PGE_2 content by ELISA (n=6 donors). Mann-Whitney U test, ##p ≤ 0.01 AC vs. MSC at the same time point.

Supplementary Table S1. List of qRT-PCR primers used in this study

Gene Name	Sequence (5'-3')
ACAN	GCACATGCCTTCTGCTT<br>GGAACCACTTGGGTCACG
BMP2	ACGAGGTCCTGAGCGAGTTC<br>GAAGCTCTGCTGAGGTGATAA
BMP6	ATTACAACAGCAGTGAATTGA<br>TTCATGTGTGCGTTGAGTG
COL2A1	TGGCCTGAGACAGCATGAC<br>AGTGTTGGGAGCCAGATTGT
COX2	TTCAAATGAGATTGTGGAAAAATTGCT<br>AGATCATCTCTGCCTGAGTATCTT
CPSF6	AAGATTGCCTTCATGGAATTGAG<br>TCGTGATCTACTATGGTCCCTCTCT
DUSP5	CTCCCACTTTCAAGAAGCAA<br>GGCAGGATCTCAGATTCGTA
FOS	TCCAGTGCCAACTTCATTCC<br>GCTGCAGCCATCTTATTCCT
FOSB	CCAGGGAAATGTTTCAGGCT<br>GAAGAGATGAGGGTGGGTTG
HNRPH1	GATGTAGCAAGGAAGAAATTGTTCAG<br>CACCGGCAATGTTATCCCAT
SOX9	GTACCCGCACTTGCACAAC<br>TCGCTCTCGTTCAGAAGTCTC
